# Taxonomic study of a new green alga, *Annulotesta cochlephila* gen. et sp. nov. (Kornmanniaceae, Ulvales, Ulvophyceae), growing on the shells of door snails

**DOI:** 10.1007/s10265-020-01239-3

**Published:** 2021-01-02

**Authors:** Noriaki Namba, Takeshi Nakayama

**Affiliations:** 1 Graduate School of Life and Environmental Sciences, University of Tsukuba, Tsukuba, Ibaraki 305-8572 Japan; 2 Faculty of Life and Environmental Sciences, University of Tsukuba, Tsukuba, Ibaraki 305-8572 Japan

**Keywords:** *Annulotesta cochlephila*, Clausiliidae, Kornmanniaceae, Phylogeny, Taxonomy, Ultrastructure

## Abstract

Some algae are known to grow on shellfish shells. Most of these have been reported in aquatic environments. The species specificity for substrate shells varies, and some algae grow only on the shells of a certain species of shellfish, such as *Pseudocladophora conchopheria* (Cladophorales, Ulvophyceae) on *Lunella coreensis* (Trochida, Gastropoda). There are very few reports of algae that grow on land snails. In this study, we discovered green algae growing on the shells of six species of door snails (Clausiliidae) from nine localities in Japan. These green algae formed a green mat composed of thalli embedded in the extracellular matrix. The thallus was composed of aggregated oval cells and peripheral branched filaments. The cells possessed a single parietal chloroplast with a pyrenoid surrounded by two starch sheaths and transversed by a thylakoid. Oil droplets in the cell and ring-like structures on the cell wall surface were frequently observed. The 18S rDNA sequences of all shell-attached algae on different clausiliid species from different localities were almost identical and formed a new clade in the family Kornmanniaceae (Ulvales, Ulvophyceae). No other algae forming visible colonies on the clausiliid shell were found. These findings indicate the presence of specificity between the alga and clausiliid shells. Based on the results of morphological observation and molecular phylogenetic analysis, we propose a new genus and new species of shell-attached green alga, *Annulotesta cochlephila.*

**Electronic supplementary material:**

The online version of this article (10.1007/s10265-020-01239-3) contains supplementary material, which is available to authorized users.

## Introduction

Symbiosis with algae is a significant event in evolution and ecosystems. Secondary endosymbioses have produced a huge diversity of photosynthetic organisms on Earth (e.g., McFadden 2001). Corals and lichens engage in symbiotic relationships with algae and play important roles in ecosystems (e.g., Gordon and Leggat 2010; Rikkinen 2003). These symbiotic relationships with algae are examples of endosymbioses—the symbionts live inside the tissues or cells of the host. On the other hand, some symbiotic algae are ectosymbionts that live on the surface of the host. Although ectosymbiotic algae have attracted less attention than endosymbiotics, various organisms, such as diatoms, dinoflagellates, red algae, mosses, angiosperms, cladocerans, copepods, isopods, opiliones, sloths, turtles, and whales, have been reported as the hosts of ectosymbiotic algae (Adams et al. 2012; Brooks 2004; Bury et al. 2015; Goff 1982; Graham et al. 2009; Hilton et al. 2013; Lindquist et al. 2005; Machado and Vital 2001; Pauli et al. 2014; Suutari et al. 2010; Tiffany 2011; Totti et al. 2010). Among these ectosymbiotic algae are the epizoic algae, which live on animals. Some epizoic algae show high specificity for the host, but some show low specificity (e.g., Gaiser and Bachmann 1994; Garbary 2010). Some epizoic algae are considered to be beneficial to the host by providing food and nutrition, protection, or camouflage against predators (e.g., Lindquist et al. 2005; Pauli et al. 2014). Some algae are known to grow on shellfish shells.

*Pyropia* (Rhodophyta) and *Monostroma* (Chlorophyta) are well known to have a shell-boring stage in their life cycle (Graham et al. 2009). *Pseudocladophora conchopheria* (Sakai) Boedeker and Leliaert (Chlorophyta) grows only on the shell of living *Lunella conreensis* (Récluz 1853), and there is no report that *P. conchopheria* grows on other substrates (Matsuyama et al. 1999). In a recent study it was suggested that a mud snail, *Bellamya chinensis* (Gray 1834), provides a hard substrate for epizoic cyanobacteria that promote the growth of the mud snail as foods (Fujibayashi et al. 2016). These symbiotic relationships between algae and species of shellfish are found in aquatic environments, and there are few reports of such relationships between algae and land snails. *Trichophilus neniae* Lagerheim is a pseudoparenchymatous green alga found on the shells of clausiliid species in South America (Lagerheim 1892). *Trypanochloris clausiliae* Geitler is a unicellular alga with irregular star-shaped cells that grow on the shells of clausiliid species in Central Europe (Geitler 1935). However, there has not been further study of either of these two relationships since the original description. We have no detailed information on the taxonomic position of these epizoic algae on land snails.

Based on our preliminary research and personal information from conchologists, we can confirm the existence of the epizoic algae on the shells of clausiliid species in Japan. To resolve the taxonomic position of the epizoic alga, we collected the epizoic algae on the shells of clausiliid species from several localities in Japan. In this study, we aim 1) to establish unialgal strains 2) to observe morphology using light and electron microscopy, 3) to construct molecular phylogenetic tree based on 18S rDNA and the internal transcribed spacer 2 (ITS-2) regions, and 4) to analyze the secondary structure of ITS-2. Finally, we propose a new genus and species, *Annulotesta cochlephila* gen. et sp. nov. for this shell-attached green alga.

## Materials and methods

### Collection, culture establishment, culturing

Clausiliid land snails covered with green algae were collected from nine locations in Japan (Table 1). The shell-attached algae were separated from the shell using a toothbrush. Unialgal cultures were then established by the micro-pipetting technique. The unialgal strains were maintained in liquid and 1.5% agar AF-6 medium (Kasai et al. 2009), Bold’s basal medium (BBM; Bischoff and Bold 1963), and 3 N-BBM + V (Kasai et al. 2009; Culture Collection of Algae and Protozoa, http://www.ccap.ac.uk) at 23 °C under white light of 10–50 µmol photons m^−2^ s^−1^ with a 14:10 h light:dark cycle. To compare the ultrastructural features of related green algae, we also studied the ultrastructure of the type strain of *Pseudendoclonium arthropyreniae* (Vischer & Klement) Tschermak-Woess (SAG 467–2). It was also cultivated under the same conditions as mentioned above.

**Table 1:** Strains used in this study

Strain	Locality	Data	Clausiliid species
NN-201	Kashiwazaki City, Niigata Prefecture	2015.6.6	*Stereophaedusa japonica* (Crosse, 1871)
NN-301	Kashiwazaki City, Niigata Prefecture	2015.6.6	*Pinguiphaedusa platydera* (Martens, 1876)
NN-401	Minamiaizu County, Fukushima Prefecture	2015.9.21	*Pinguiphaedusa platydera* (Martens, 1876)
NN-501^a^	Hitachiota City, Ibaraki Prefecture	2015.6.13	Unknown
NN-601	Toyohashi City, Aichi Prefecture	2015.6.20	*Pinguiphaedusa kubinaga* (Kuroda, 1936)
NN-701	Shizuoka City, Shizuoka Prefecture	2015.10.10	Unknown
NN-901^b^	Hamamatsu City, Shizuoka Prefecture	2015.10.11	*Mesophaedusa mikawa* (Pilsbry, 1905)
NN-T1^c^	Chichibu City, Saitama Prefecture	2015.7.24	*Pinguiphaedusa platydera* (Martens, 1876)
NN-OKN1A	Nago City, Okinawa Prefecture	2016.9.19	*Nesiophaedusa praeclara* (Gould, 1859)
NN-SIG1^d^	Maibara City, Shiga Prefecture	2016.12.1	*Tyrannophaedusa mikado* (Pilsbry, 1900)

^a^The sample was collected by Mr. Yasuhiko Chikami

^b^The sample was collected by Dr. Hirotaka Nishi

^c^The sample was collected by Dr. Yosuke Degawa

^d^The sample was collected by Dr. Takahiro Hirano and Dr. Takumi Saito

### Light microscopy

The shells with algae were observed using a fluorescence stereomicroscope multi-stand system, VB-S20 (Keyence, Osaka, Japan). Light microscopy of unialgal cultures was carried out using a Zeiss Axio Imager A2 microscope (Zeiss, Oberkochen, Germany) equipped with Nomarski differential interference contrast (DIC) optics and an Olympus IX71 inverted microscope (Olympus, Tokyo, Japan). Microphotographs were taken with an Olympus DP-71 CCD camera (Olympus, Tokyo, Japan) and a digital single-lens reflex camera (Canon EOS 70D, Canon, Tokyo, Japan).

### Electron microscopy

For scanning electron microscopy (SEM), the cells of NN-301 were placed on a glass plate coated with 0.1% (w/v) poly-L-lysine (Wako chemical, Japan), fixed with 2.5% glutaraldehyde (GA) in 0.1 M cacodylate buffer (pH 7.2), and stored at room temperature for 1 h. Fixed cells were washed three times with 0.2 M cacodylate buffer. Cells were postfixed in 1% osmium tetroxide at room temperature for 1 h and then washed three times with the same buffer. Dehydration was performed using a graded series of 30%–100% ethanol (v/v). After dehydration, cells were placed in a 1:1 mixture of 100% ethanol and t-butyl alcohol for 20 min and t-butyl alcohol for 20 min twice. The cells were then freeze-dried using a VFD-21S vacuum freeze dryer (Vacuum Device Co., Ltd., Ibaraki, Japan) and coated with platinum-palladium using a Hitachi E-102 sputter-coating unit (Hitachi High-Technologies Corp., Tokyo, Japan). Observations were made using a JSM-6330F field emission scanning electron microscope (JEOL, Ltd., Tokyo, Japan).

The shell of a snail collected from Kashiwazaki City, Niigata Prefecture, on June 6, 2015, was broken, and the fragments were fixed and prepared for SEM as described above.

For transmission electron microscopy (TEM), the cultivated cells were centrifuged and fixed for 2 h at 4 °C with a mixture of 2.5% GA in 0.1 M cacodylate buffer (pH 7.2). Fixed cells were washed with 0.2 M cacodylate buffer three times. Post-fixation was performed using two methods: 1% osmium tetroxide at 4 °C for 1 h and 6% potassium permanganate aqueous solution at room temperature for 15 min. Fixed cells were washed with 0.2 M cacodylate buffer three times. Dehydration was performed using a graded series of 30%–100% ethanol (v/v). After dehydration, cells were placed in a 1:1 mixture of 100% ethanol and acetone for 10 min and acetone for 10 min twice. The cells were embedded in resin by placement in a 1:1 mixture of acetone and Agar Low Viscosity Resin R1078 (Agar Scientific Ltd., Stansted, U.K.) for 30 min and resin for 1 h, twice. The resin was polymerized by heating at 70 °C for 12 h. For the holotype specimen, resin-embedded cells were mounted on glass slides and polymerized under the same conditions. Ultrathin sections were prepared on a Reichert Ultracut S ultramicrotome (Leica, Vienna, Austria) and double-stained with 2% (w/v) uranyl acetate and lead citrate (Sato 1968). Transmission electron microscopy was performed using a Hitachi H-7650 electron microscope (Hitachi High-Technologies Corp., Tokyo, Japan) equipped with a Veleta TEM CCD camera (Olympus Soft Imaging System, Münster, Germany).

### DNA extraction, polymerase chain reaction, and sequencing

Total DNA was extracted from both algal mats on the shell and cultivated using the DNeasy Plant Mini Kit (Qiagen, Hilden, Germany). After adding 400 μm of Buffer AP1 and 4 μL RNase A, a small quantity of glass beads was added and vortexed to break the cells. The following steps were performed according to the manufacturer’s instructions. The 18S rDNA was amplified by polymerase chain reaction (PCR) using KOD Fx Neo (Toyobo, Osaka, Japan) with SR1 and SR12 primers (Nakayama et al. 1998). For ITS rDNA, SR10 (Nakayama et al. 1998) and ITS055R primers (Marin et al. 2003) were used. PCR amplifications included initial denaturation at 94 °C for 2 min, followed by 30 cycles of denaturation at 98 °C for 10 s, annealing at 53 °C for 30 s, and extension at 68 °C for 90 s. PCR products were checked by electrophoresis and excised. Purification of PCR products was carried out using the QIAquick Gel Extraction kit (Qiagen, Hilden, Germany). A-tailing was then performed at 70 °C for 20 min using TaKaRa EX tap (Takara Bio, Shiga, Japan) with dATP and then cloned into the pGEM^®^ T-easy vector (Promega, Tokyo, Japan) because intra-strain polymorphism in the ITS sequences of shell-attached algae was detected by direct sequencing. Plasmid DNA was extracted and purified using QIAprep Miniprep (Qiagen, Hilden, Germany). DNA sequencing was carried out using BigDye Terminator v. 3.1 cycle sequencing kit (Applied Biosystems, Tokyo, Japan) and a 3130 Genetic Analyzer (Applied Biosystems, Tokyo, Japan). The accession numbers of 18S rDNA and ITS rDNA sequences are LC577562–LC577604.

### Phylogenetic analysis

The 18S rDNA sequences of 50 ulvophyceans from GenBank (Table S1) and the sequences acquired in this study were aligned using MAFFT v7.409 (Kathoh and Standley 2013). The gaps and poorly aligned regions of the alignment were excluded by trimAl v1.2 (Capella-Gutiérrez et al. 2009), and the alignment was checked using MEGA7 (Kumar et al. 2016). The alignment file is deposited in figshare (10.6084/m9.figshare.13072178). Phylogenetic analyses were performed using maximum likelihood (ML) and Bayesian methods. In ML analysis, a phylogenetic tree was constructed using IQ-TREE Ver. 1.6.7 (Nguyen et al. 2015) with the TIM3e + R4 model that decided by automatic estimation in IQ-TREE. Bootstrap values were calculated using the standard nonparametric bootstrap method with 100 replicates. Bayesian analyses were performed using MrBayes v3.2.6 (Ronquist et al. 2012) with the SYM Gamma model. One cold and three heated Markov chain Monte Carlo (MCMC) chains were run for 1,000,000 generations, with sampling every 500 cycles. The initial 250,000 cycles were discarded as burn-in. Bayesian posterior probability was calculated from the remaining trees. The alignment data for the ITS-2 sequences of *A. cochlephila* were generated using the same method mentioned above. The ML tree was constructed using the IQ-TREE Ver. 1.6.7 with the TPM2 + F + I model that decided by automatic estimation in IQ-TREE. Bootstrap values were calculated using the same conditions as noted above. In this analysis, 19 clone sequences were used and not specified roots.

### ITS-2 secondary structure

ITS-2 regions of all strains of *A. cochlephila* were annotated using a web interface in the ITS2 database (Ankenbrand et al. 2015; http://its2.bioapps.biozentrum.uni-wuerzburg.de/). The sequences of ITS-2 were folded as described previously (Matsuzaki et al. 2014) using Centroidfold (Hamada et al. 2009) and RNAfold at the RNAfold WebServer (Gruber et al. 2008; http://rna.tbi.univie.ac.at/cgi-bin/RNAWebSuite/RNAfold.cgi). The sequences of the ITS-2 regions were aligned and the existence of compensatory base changes (CBCs) was confirmed using the program 4SALE version 1.7.1 (Seibel et al. 2006). The ITS-2 secondary structure of *A. cochlephila* was drawn using VARNA version 3.93 (Darty et al. 2009). For the comparison among the strains of *A. cochlephila*, the ITS-2/CBC approach was applied following the procedure described by Darienko et al. (2017). The alignments of the conserved region of ITS-2 have been translated into base pair alignment by using a number code for each base pair (1 = A-U; 2 = U-A; 3 = G-C; 4 = C-G; 5 = G•U; 6 = U•G), and the resulting numeric codes of each sequence are given in Fig. S1.

## Results

### Light microscopy

**Fig. 1:**
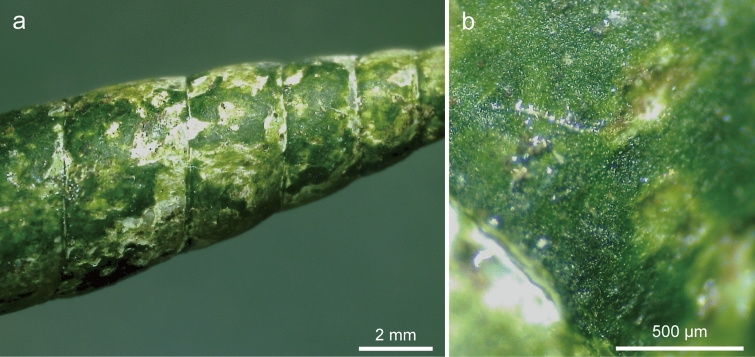
Stereomicrographs of the green mat of *Annulotesta cochlephila* gen. et sp. nov. on the shell of *Pinguiphaedusa platydera*. The green mat-like structure forms the surface of the shell. **a** Global image. **b** Enlarged image

**Fig. 2:**
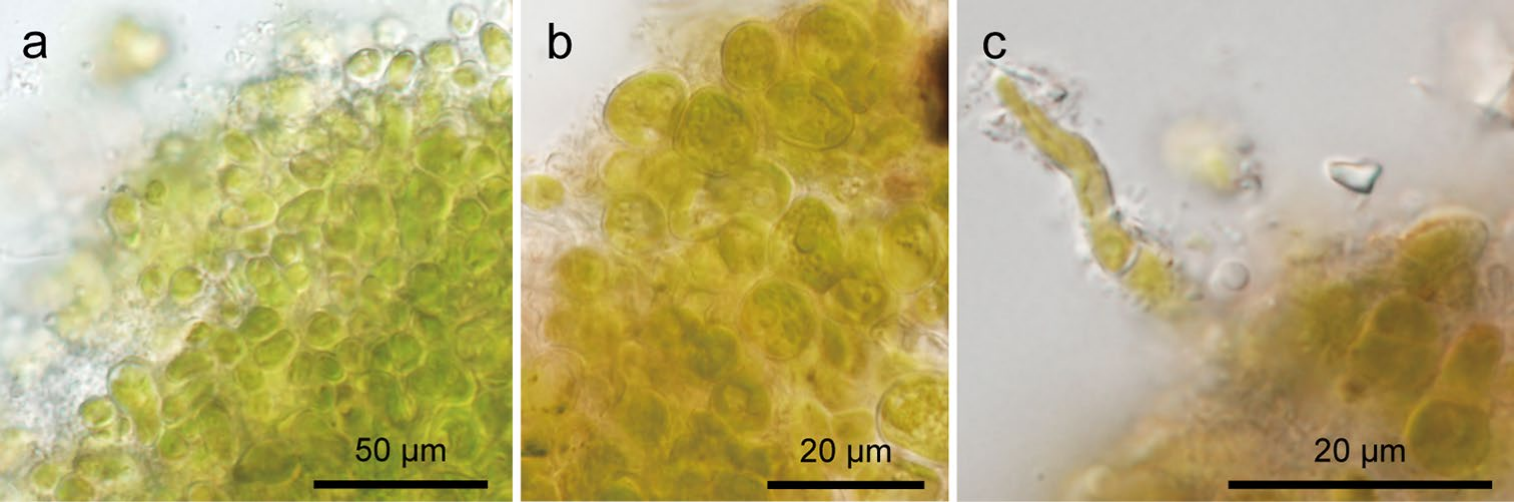
Light micrographs of *A. cochlephila* gen. et sp. nov. scratched off from the shell surface of *P. platydera*. **a, b** Elliptical to spherical cells congregated tightly. **c** Filament composed of elongated cells

Shell-attached algae formed a distinct green mat on the surface of the shells of clausiliid snails (Fig. 1a, b). When the green mat cut off with a razor was observed under a light microscope, many elliptical to spherical cells, forming a tightly congregated mat, could be seen (Fig. 2a, b). Filaments composed of elongated cells were occasionally observed (Fig. 2c). However, it was difficult to distinguish by morphological characteristics alone whether these filaments were a single species or the complex of multiple species, because the size and shape of cells varied in this state.

**Fig. 3:**
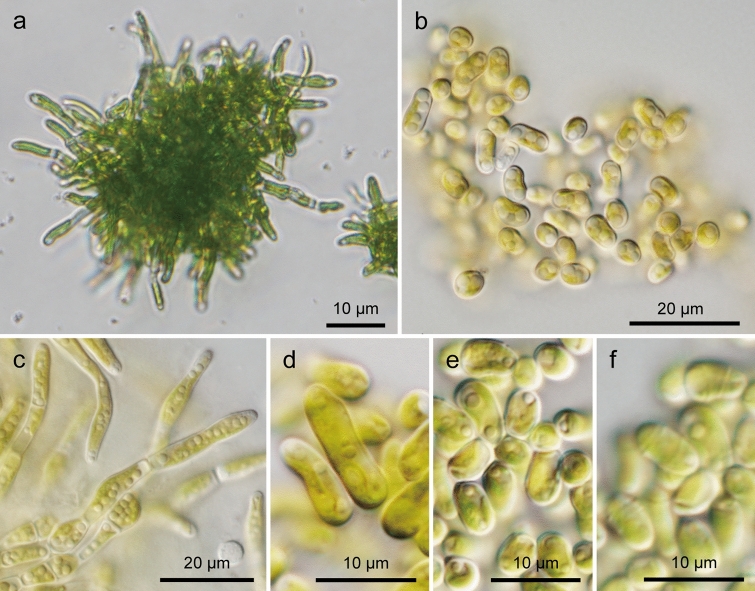
Light micrographs of *A. cochlephila* gen. et sp. nov. strain NN-301. **a** Typical thallus with peripheral radiating filaments. **b** Elliptical to spherical cells composing the central part of a thallus. **c** Filaments radially extending from thallus. Cells containing many oil droplets (arrows) **d** A cell with a pyrenoid (Py). **e** Oval cells with large oil droplets (arrows). **f** Surface view of oval cells showing ring-like structures (arrowheads)

In the isolated strains cultured on agar medium for several weeks, shell-attached algae formed a pseudoparenchymatous thallus (Fig. 3a). In the developed thallus, the central part was composed of oval cells that were 5–10 μm long and 3–5 μm wide (Fig. 3b). The peripheral part of the thallus formed radially extending uniseriate filaments composed of cylindrical cells that were 15–23 μm long and 3–5 μm wide (Fig. 3c). These filaments were rarely branched. The cells constituting the filament were firmly attached to each other, but the central part of the thalli was fragile and easily disintegrated by the preparation on a glass slide. Each cell was surrounded by a cell wall and had a parietal chloroplast (Fig. 3b, c). The pyrenoid was sometimes observed in the chloroplast (Fig. 3d), but it was frequently undetectable. Many cells also possessed one to many oil droplets (Fig. 3c, e). A few ring-like structures were observed on the cell wall of the cells in the central part (Fig. 3f). In the strains cultured in liquid medium, the thallus formed peripheral filaments only in the early stage, and elliptical to short cylindrical cells were densely packed (Fig. 3b). The thallus did not firmly stick to the substratum. Zoospores and resting spores were not observed under any culture conditions. The morphological features mentioned above were common to all shell-attached algal strains studied.

### Scanning electron microscopy

**Fig. 4:**
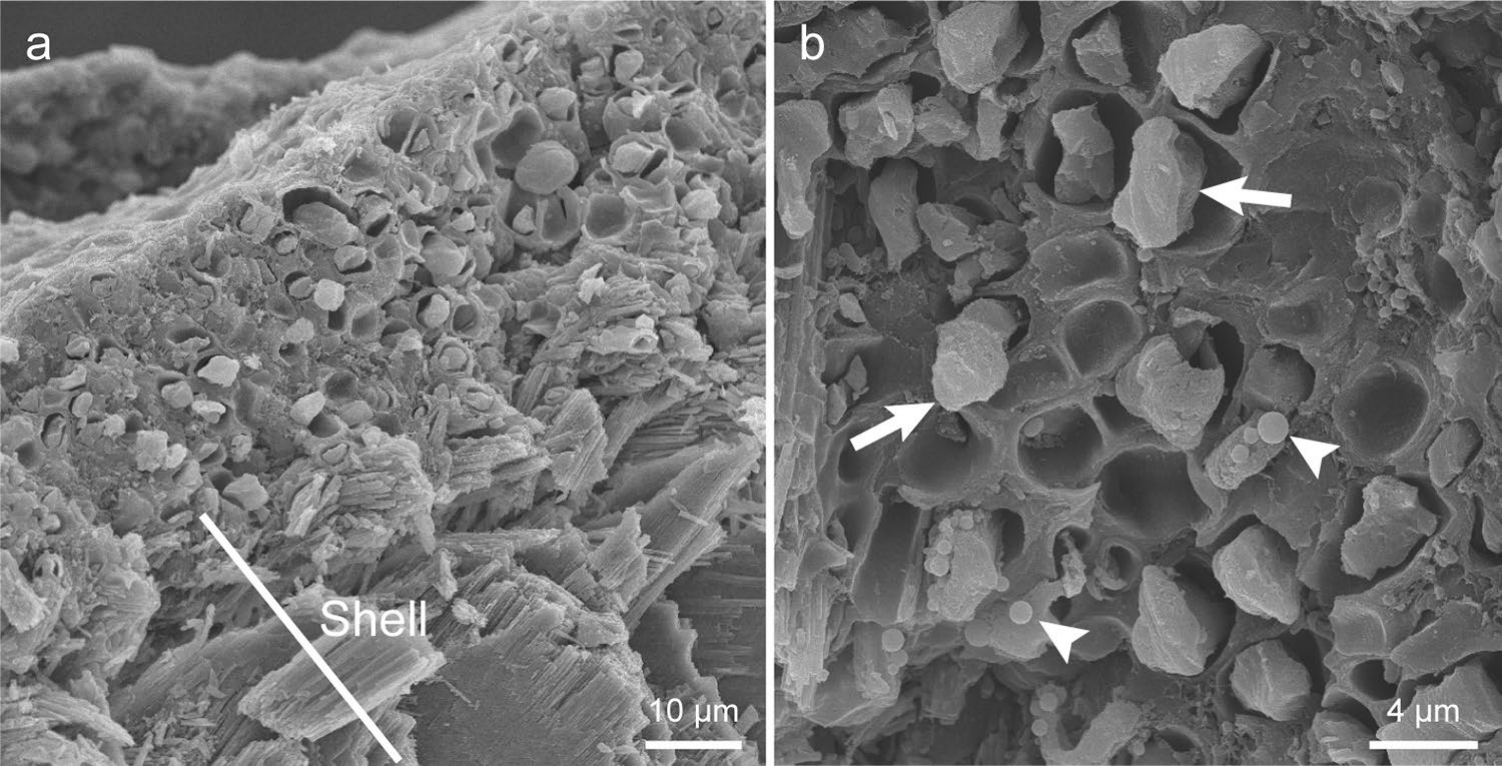
Scanning electron micrographs of a mat on the shell of *P. platydera*. **a** Transverse section of a mat tightly attached to the shell surface. **b** Enlarged image of a mat showing possible cells of *A. cochlephila* gen. et sp. nov. (arrows) and bacteria-like structure (arrowheads)

A piece of shell with a green mat was observed using a scanning electron microscope. The outer surface of the limestone layer of the shell was covered with a homogeneous matrix approximately 20–30 μm thick (Fig. 4a). The surface of the matrix was smooth. In the matrix, there were many oval structures with a diameter of approximately 5 μm, which probably represented the cells of shell-attached algae, and there were also many holes that were probably the trace of fallen cells (Fig. 4a, b). Smaller spherical to rod-shaped structures that probably represented bacteria were also observed (Fig. 4b).

**Fig. 5:**
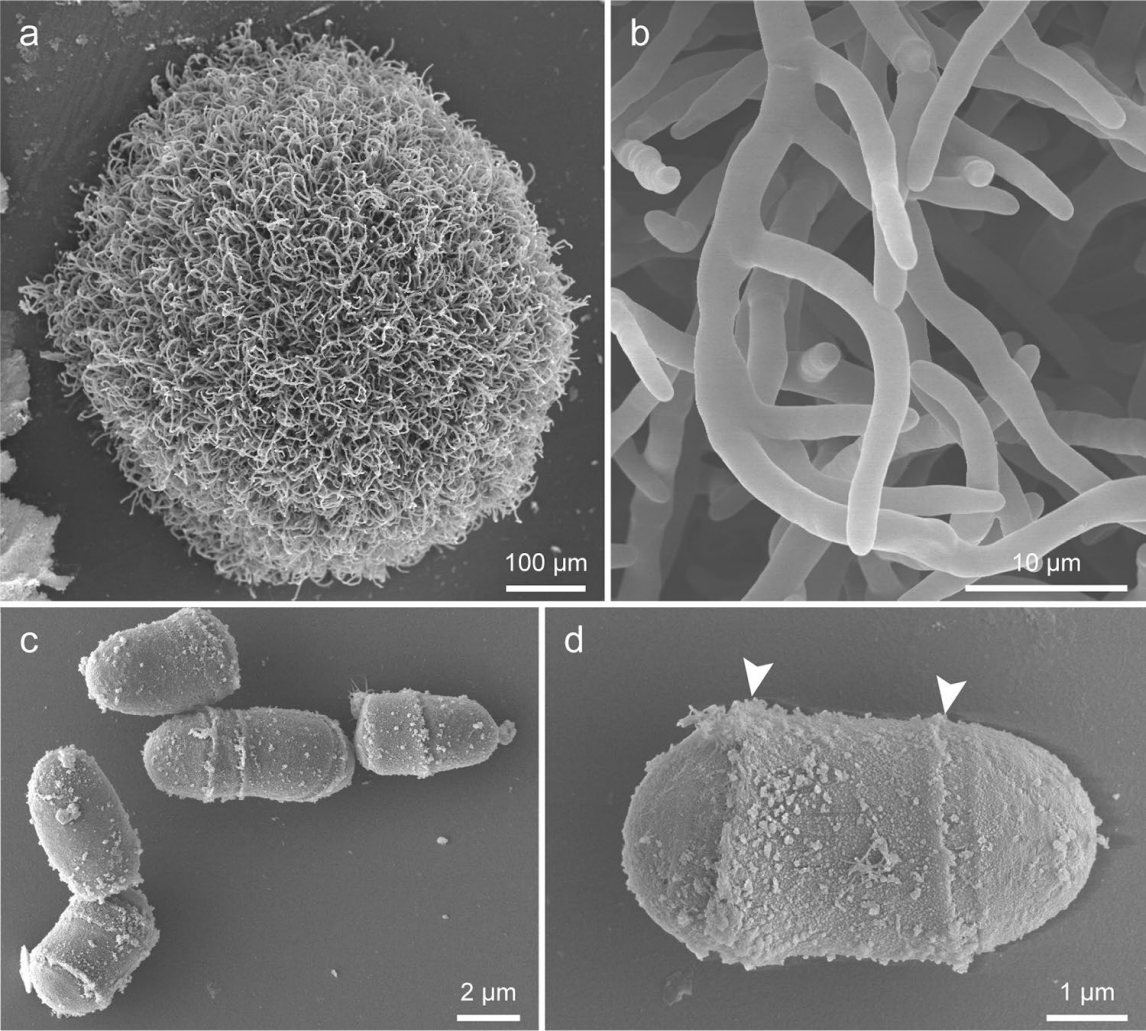
Scanning electron micrographs of *A. cochlephila* gen. et sp. nov. strain NN-301. **a** Global image of a thallus cultivated on agar media **b** Peripheral branching filaments **c, d** Oval cells with ring-like structures (arrowheads)

The strains cultured on agar media formed dome-shaped thalli with radially extending filaments at the surface (Fig. 5a). The filaments branched infrequently, and no regularity was found in the direction and number of branches (Fig. 5b). The junction of cells in a filament was not detected (Fig. 5b). This feature is probably due to the presence of an extracellular matrix covering the cell surface. The sample cultured in liquid medium consisted of elliptical cells, and firm adhesion between cells was not observed (Fig. 5c). One to a few ring-like structures encircling the surface of the cell wall were frequently observed (Fig. 5c, d).

### Transmission electron microscopy

**Fig. 6:**
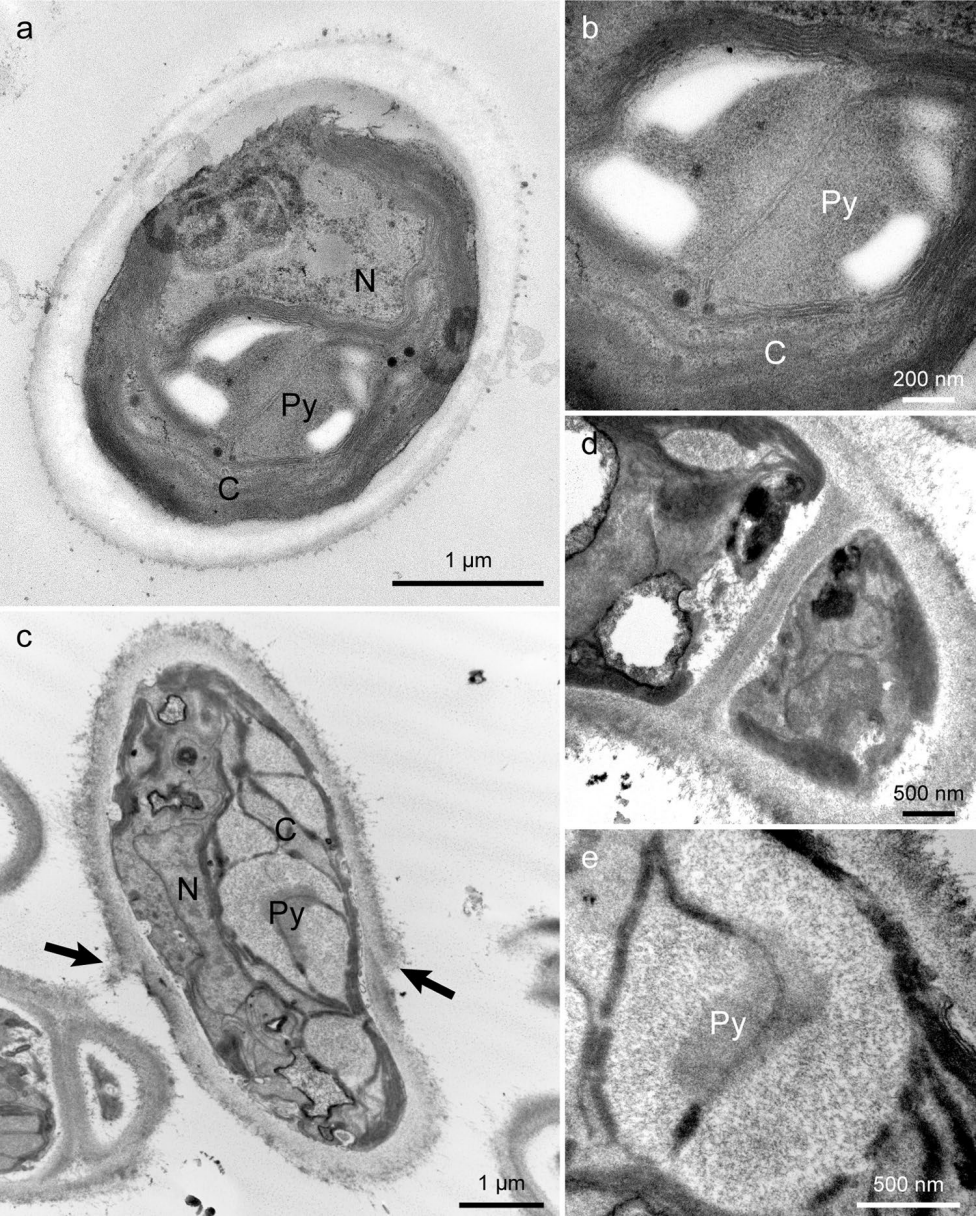
Transmission electron micrographs of *A. cochlephila* gen. et sp. nov. strain NN-301 fixed by OsO4 (**a, b**) and KMnO4 (**c–e**). **a, c** Global image of a cell. Arrow in **c** indicates a ring-like structure on the cell wall. **b, e** A pyrenoid traversed by a single thylakoid. **d** Cross wall between cells. *C*, chloroplast; *N*, nucleus; *Py*, prenoid

The cell of shell-attached algae was surrounded by a thick (ca. 250–500 nm) cell wall (Fig. 6a, c). The cell wall was roughly differentiated into an inner, less electron-dense layer and an outer, fibrous layer. In the probable filamentous part of the thallus, the neighboring cells shared the outer layer of the cell wall (Fig. 6d). Some sections showed that the outer part of the cell wall was turned up (Fig. 6c). This structure probably represents the ring-like structure on the cell wall found in light and scanning electron microscopy. The cell contained a chloroplast, mitochondria, and a nucleus (Fig. 6a, c). The chloroplast possessed irregularly layered thylakoid lamellae, starch grains, and a pyrenoid (Fig. 6a, c). The pyrenoid matrix was surrounded by two saucer-shaped starch sheaths and transversed by a single thylakoid membrane (Fig. 6b, e).

**Fig. 7:**
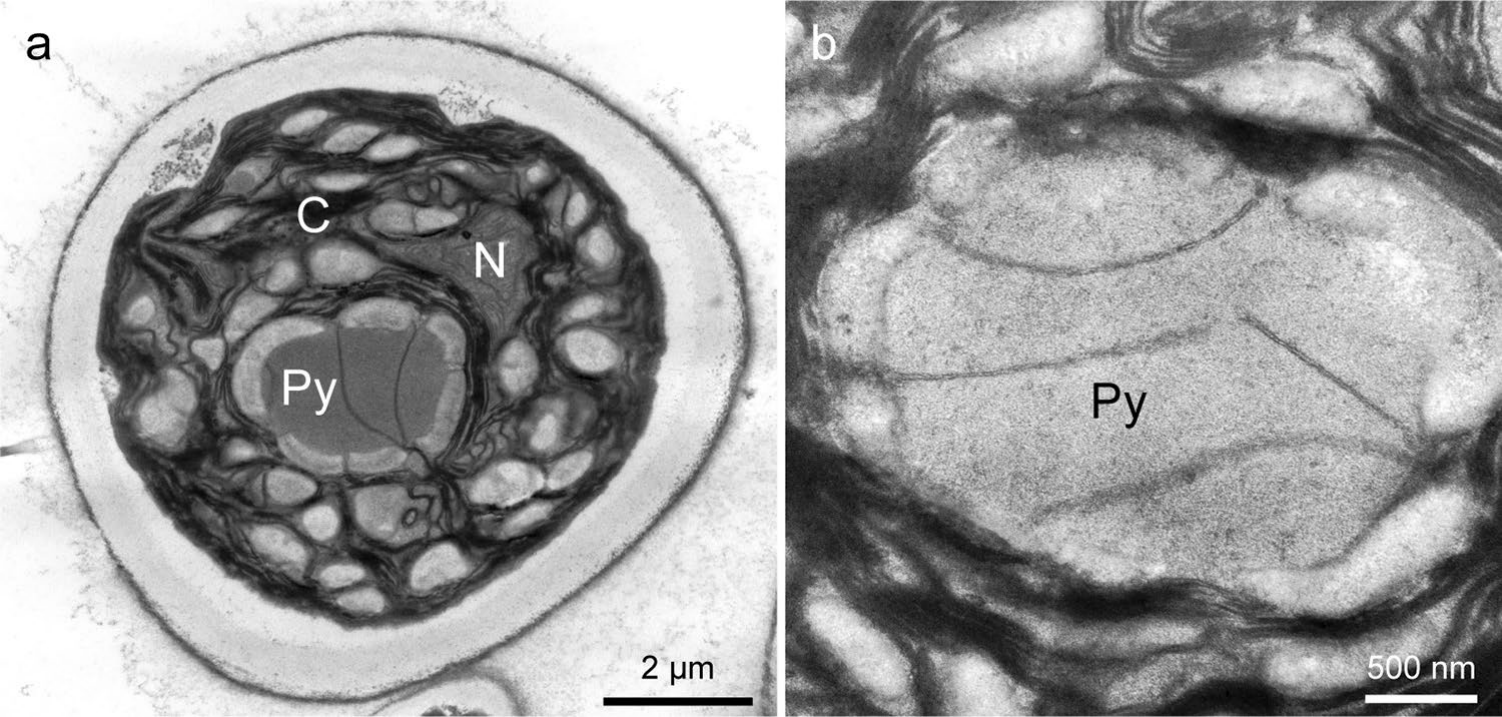
Transmission electron micrographs of *P. arthropyreniae* fixed by KMnO4. **a** Global image of a cell. **b** Pyrenoid traversed by three thylakoids. *C*, chloroplast; *N*, nucleus; *Py*, prenoid

The cell of *Pseudendoclonium arthropyreniae* was also surrounded by a thick (ca. 500 nm) cell wall (Fig. 7a). The cell wall was differentiated into a thick, inner translucent layer and an outer, thin electron-dense layer, and was surrounded by fibrous material. The turning up of the cell wall observed in the previously described sample (Fig. 6c) was not evident in *P. arthropyreniae*. The cell contained chloroplasts, mitochondria, and a nucleus (Fig. 7a). The chloroplast possessed a pyrenoid surrounded by many starch grains, and the matrix was transversed by three separated thylakoid membranes (Fig. 7b).

### Phylogenetic analysis of the 18S rDNA

**Fig. 8:**
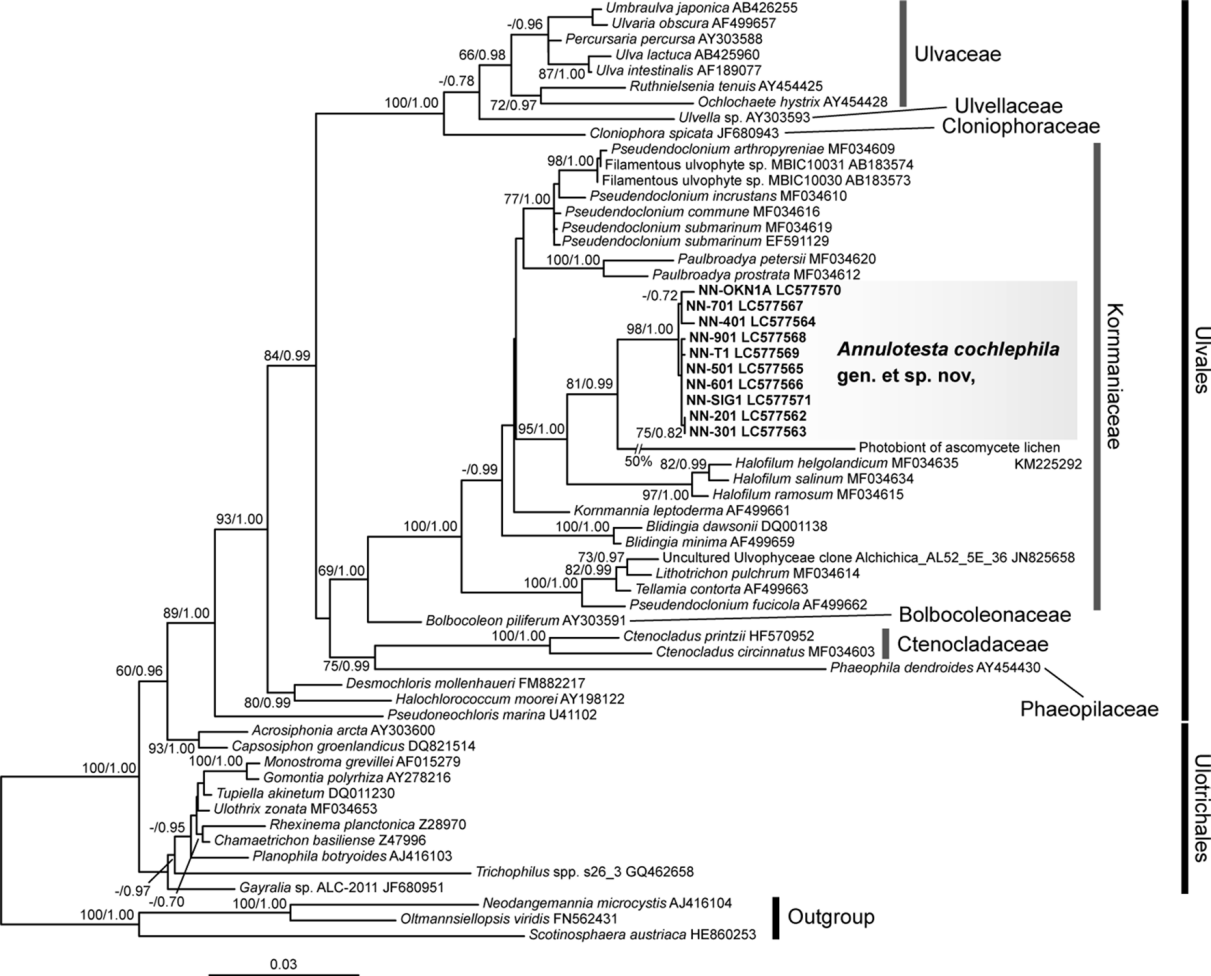
Maximum-likelihood (ML) phylogenetic tree of the Ulvophyceae using 1656 positions from the 18S rDNA sequences. Values on each node indicate ML bootstrap probabilities (≥ 60%)/Bayesian posterior probabilities (≥ 0.7)

The 18S rDNA sequences from the green mat on the shell of the living clausiliid land snail and the isolated unialgal strains were completely identical for each sample listed in Table 1. The sequences from these samples showed high similarity (98%). The BLAST search based on these sequences found no identical sequences even in the environmental sequences, but showed high similarity to the sequences of the Ulvales (Ulvophyceae). Therefore, phylogenetic analysis of the ulvalean algae, including shell-attached algae, was performed (Fig. 8). The shell-attached algae formed a robust clade with high statistical support [bootstrap value (BV) = 98%, Bayesian posterior probability (BPP) = 1.00]. This clade was included in the Kornmanniaceae (Ulvales) with maximum statistical support (BV = 100%, BPP = 1.00). Within the Kornmanniaceae, the shell-attached algal clade was sister to the photobiont of an ascomycete lichen (*Acremonium stroudii* K. Fletcher, F.C. Küpper & P. van West) collected from the splash zone on Ascension Island (Fletcher et al. 2017) (BV = 81%, BPP = 0.99). This clade was sister to *Halofilum,* with high statistical support (BV = 95%, BPP = 1.00). *Pseudendoclonium,* including *P. arthropyreniae,* was distantly related to the shell-attached algal clade in the Kornmanniaceae.

### Analysis of rDNA ITS-2

**Fig. 9:**
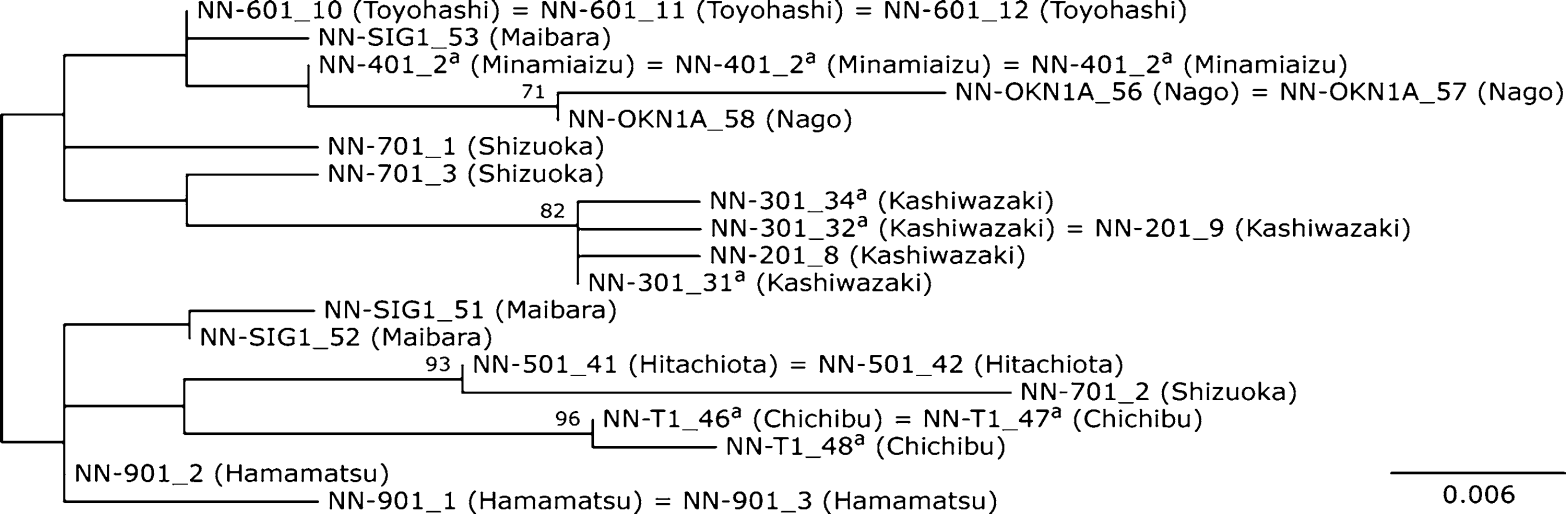
Unrooted maximum likelihood trees of 19 clone sequences of ITS-2 regions (242 positions) from *A. cochlephila* gen. et sp. nov. Values on each node indicate ML bootstrap probabilities (≥ 60%). ^a^Host species is *P. platydera*. The locality of the sample is in parenthesis (city)

To verify the relationship among shell-attached algae, host clausiliid species, and habitat, we performed a phylogenetic analysis using sequences of shell-attached algae was and based on ITS-2 sequences (Fig. 9). The non-rooted phylogenetic tree showed that the shell-attached algae collected in the same place (e.g., strain NN-301 and NN-201) formed a clade (BV = 80%). However, the algae isolated from the clausiliid land snails in the same prefecture (Shizuoka) were not closely related. Moreover, the shell-attached algae isolated from the same host clausiliid species (e.g., NN-301, NN-401, and NN-T1) were placed at different phylogenetic positions.

Intra-strain polymorphism in the ITS of shell-attached algae was detected by direct sequencing. The sequencing of clonal PCR products from selected strains revealed that substitutions and indels (insertion-deletion mutations) were present in ITS. However, the prediction of the secondary structure showed that such polymorphism did not affect the secondary structure of ITS-2. The predicted secondary structure of ITS-2 in shell-attached algae possessed four helices, as in other chlorophytes (Caisová et al. 2013; Mai and Coleman 1997), and no compensatory base changes (CBCs) were detected among the strains of shell-attached algae (Fig. S1). However, only two Hemi CBCs were discovered in helices II and III.

## Discussion

### Taxonomy of shell-attached algae

To the best of our knowledge, only two species, *Trypanochloris clausiliae* and *Trichophilus neniae*, have been reported as epizoic algae on the shells of clausiliid land snails (Geitler 1935; Lagerheim 1892). The shell-attached alga discovered in the present study is similar to *Tc. clausiliae* and *Tp. neniae*, having a single parietal green chloroplast and no distinct pyrenoid under light microscopy. However, *Tc. clausiliae* is a star-shaped unicellular alga with bifurcate arms extending radially and develops into a large sporangium up to 70 × 30 μm (Geitler 1935). Thus, *Tc. clausiliae* is clearly different from the shell-attached alga found in the present study. *Trichophilus neniae* is a filamentous alga forming pseudoparenchymatous thalli, as does the alga described in the present study. However, *Tp. neniae* differs in its degree of penetration into the shell and the production of zoospores and does not have a ring-like structure on the cell wall (Lagerheim 1892). Furthermore, the sequence of *Trichophilus* sp. that are probably closely related with the type species of *Trichophilus*, *T. welckeri* Weber Bosse 1887, because of their unique common habitat (on sloth hairs) is distantly related to the sequences of shell-attached alga in the present study (Fig. 8). Therefore, we concluded that no alga identical to the shell-attached algae found in the present study has been described.

The shell-attached alga in the present study is morphologically similar to some members of the Kornmanniaceae, such as *Pseudendoclonium* (including *Dilabifilum*), *Paulbroadya*, *Lithotrichon,* and *Halofilum*. These green algae are reported from marine and freshwater environments or associated with lichens and frequently form pseudoparenchymatous thalli with branched filaments (Darienko and Pröschod 2017; John and Johnson 1989; Johnson and John 1990; Mullins 2007). The shell-attached alga also possesses pseudoparenchymatous thalli with marginal branched filaments. Because of their high morphological plasticity, the morphological distinctions of these green algae are not clear (Darienko and Pröschod 2017). However, the ring-like structure found in the shell-attached algae has not been reported for these algae (Darienko and Pröschod 2017; John and Johnson 1989; Johnson and John 1990; Mullins 2007). Although ultrastructural studies of these algae are scarce, the present study indicates that the ultrastructure of pyrenoids in shell-attached algae is different from that of *Pseudendoclonium arthropyreniae*. Furthermore, the phylogenetic analysis based on 18S rDNA indicated that the shell-attached algae form a clade that is phylogenetically separable from the other branching filamentous green algae. Some filamentous green algae possessing similar morphology have been described without molecular data (e.g. *Gongrosira* spp.). However, these green algae are also different from the shell-attached algae in some morphological features such as the absence of ring-like structure on cell wall (e.g. Johnson and John 1992).

The shell-attached algae collected from different species of clausiliid land snails and different localities in Japan show no morphological differences. Although some differences were found in the sequences of 18S rDNA and ITS, we found no clear groupings in the shell-attached algae. Furthermore, CBCs were not detected in the rDNA ITS-2 of these algae.

Based on the considerations mentioned above, we propose a new genus, *Annulotesta*, in the Kornmanniaceae, Ulvales, Ulvophyceae, for the shell-attached algae found in the present study. In addition, we classify all these algae into a single species, *Annulotesta cochlephila*. However, this taxonomic treatment is tentative. Additional morphological and molecular studies based on a large number of samples collected globally are necessary to establish the precise species classification system of *Annulotesta.*

#### Diagnosis

##### *Annulotesta* Namba & T. Nakayama gen. nov.

Diagnosis: Pseudoparenchymatous alga composed of central rounded and peripheral cylindrical cells on solid media. Cylindrical cells form branching filaments. Vegetative cells have a parietal plate-like chloroplast with a pyrenoid. One to a few ring-like structures were situated on the cell wall of the cells in the central part. The genus differs from similar genera by 18S rDNA sequences.

Type species (designated herein): *Annulotesta cochlephila* Namba & T. Nakayama sp. nov.

##### *Annulotesta cochlephila* Namba & T. Nakayama sp. nov.

Diagnosis: The central part of the thallus is composed of rounded cells (5–10 × 3–5 μm). Peripheral filaments extending radially are composed of cylindrical cells (15–23 × 3–5 μm). No regularity is found in the direction and number of branching. Filaments are rare and short in liquid media. Cells contain a parietal plate-like chloroplast with a pyrenoid surrounded by starch plates. Matured cells contain oil droplets. Zoospores and sexual reproduction are not observed.

Habitat: The shell surface of land snails, family Clausliidae.

Type locality: Kashiwazaki City, Niigata Prefecture, Japan.

Holotype: A permanent microscope slide; TNS–AL–58974 s in TNS (Department of Botany, National Museum of Nature and Science).

Etymology: The generic name refers to the cell wall possessing a ring-like structure (*annulatus* = ring-like pattern, *testa* = envelope), and the specific epithet refers to its habitat, the shell surface of land snails, family Clausliidae (*cochlea* = snail, *philus* = loving).

In the molecular phylogenetic analysis based on 18S rDNA, *Annulotesta* is most closely related to the unidentified photobiont of ascomycete lichen found on Ascension Island (Fletcher et al. 2017). The morphological observation of this photobiont is insufficient, and its taxonomic assignment requires further study. Among the described genera, *Halofilum* is the most closely related to *Annulotesta* in the molecular phylogenetic analysis.

### Specificity of *Annulotesta cochlephila* and clausiliid land snails

In the present study, we showed high specificity in the epizoic relationship between *A. cochlephila* and clausiliid land snails. Although not all shells of the sampled clausiliid land snails were covered with visible algal colonies, all algae forming visible colonies on the shells were the same species, *A. cochlephila*. We did not find *A. cochlephila* in other habitats, such as the surfaces of rocks, logs, or soil, even in the locality where the clausiliid land snails with algal colonies were collected. The possibility that the main habitat of *A. cochlephila* is other than the shell of clausiliid land snails cannot be excluded, and further studies (for example, environmental DNA surveys) are necessary to clarify this. However, it should be noted that the algae forming visible colonies on the shells of the clausiliid land snails shell are exclusively *A. cochlephila*, including shells of different species and shells collected from different localities.

Interestingly, we found no other land snails with *A. cochlephila* colonies than the clausiliid land snails, even in the localities where *A. cochlephila* was found. Furthermore, the hosts of two known epizoic algae (*Trypanochloris clausiliae* and *Trichophilus neniae*; see above) are also exclusively clausiliid land snails (Geitler 1935; Lagerheim 1892). This evidence suggests that the shell of the clausiliid land snails is more suitable as a substrate for shell-attached algae in comparison to the shells of other land snails. It has been reported that some land snails (e.g., *Euhadra*) have minute-scale roughness on the surface of the shell, and it is thought that this structure prevents the adhesion of dirt on the shell by holding water molecules (Isu 2009). This type of shell surface might make it difficult for epizoic algae to adhere to the shell; in contrast, species of clausiliid land snails might lack this structure.

We cannot find evidence of coevolution of *A. cochlephila* and the clausiliid land snail species. The strains isolated from *Pinguiphaedusa platydera* (Martens 1876) in different localities (Niigata, Saitama, and Hukushima) were not closely related in the phylogenetic tree. On the other hand, the strains isolated from different species [*P. platydera* and *Stereophaedusa japonica* (Crosse 1871)] in the same locality (Niigata) showed nearly the same sequences. However, the algae isolated from the clausiliid land snail species in the same prefecture (Shizuoka) were not closely related. Thus, there appears to be no clear correlation between the phylogeny of *A. cochlephila* and geographical location.

The specificity of epizoic algae and their hosts varies. Some epizoic algae show high specificity for their host. *Pseudocladophora conchopheria* (Ulvophyceae, Cladophorales) exhibits very high specificity and grows only on the shell of *Lunella conreensis* (Récluz 1853) (Matsuyama et al. 1999). The epizoic algae on sloths, *Trichophilus welckeri* (Ulvophyceae, Ulotrichales), is the main epizoic algae on sloth hairs, and the alga is not found in other habitats (Pauli et al. 2014; Suutari et al. 2010). *Trichophilus welckeri* grows in various species of sloths, as in the case of *A. cochlephila*. However, the genetic variation of *T. welckeri* corresponds with the genus of sloths, which suggests coevolution of *T. welckeri* and sloths (Suutari et al. 2010). Some diatoms (e.g., *Plumosigma*, *Bennettella*, *Epipellis*, *Epiphalaina*, *Tursiocola*, and *Tripterion*) have been found specifically on the surface of large marine animals such as whales, manatees, and sea turtles (Frankovich et al. 2015; Majewska et al. 2017; Tiffany 2011). However, no distinct correlation between animal species and epizoic diatom species has been found (Frankovich et al. 2015; Holmes 1993). We have not found an epizoic relationship that has the degree of specificity shown in *A. cochlephila* and clausliid land snails, and this relationship is a new example of the epizoic relationship between algae and animals. Because the land snails show a high topical specification rate due to their low motility (e.g., Chiba 1999; Phillips et al. 2020; Schilthuizen et al. 2006), a detailed study on the genetic variation of *A. cochlephila* is an interesting topic for future research.

### Relationship between *Annulotesta cochlephila* and clausiliid land snails

*Annulotesta cochlephila* attaches to the shell by burying in the extracellular matrix that is tightly adhered to the shell surface. The algae did not penetrate into the limestone layer of the shell. On the other hand, *Trichophilus neniae* and *Pseudocladophora conchopheria* penetrate into the shell (Lagerheim 1892; Matsuyama et al 1999). In addition, some species of cyanobacteria (*Cyanosacus*, *Hyella*, *Leptolyngbya*) and green algae (*Gomontia*, *Ostreobium*, *Phaeophila*) are also known as shell-boring algae of marine shellfish (Pantazidou et al. 2006). The origin and components of the extracellular matrix on shells are currently uncertain. It is unknown whether the matrix is produced by *A. cochlephila* alone or as a result of coexistence with other organisms such as bacteria. No extracellular matrix has been observed around *A. cochlephila* under culture conditions, which suggests that at least some organisms are necessary to form the matrix. In addition, the relationship between *A. cochlephila* and the periostracum (the proteinaceous outer sheath of many molluscan shells) is not clear at present, and further study is necessary to understand the extracellular matrix on the shell.

The ecological significance of algal adhesion on the clausliid land snail shell is interesting but poorly understood at present. In the case of cyanobacterial colonization on the shells of mud snails, a mutualistic relationship has been suggested (Fujibayashi et al. 2016). Cyanobacteria have access to hard substrates, the shells, in paddy fields where only soft substrates are present. Mud snails cannot feed on cyanobacteria that grow on their own shells, but they can feed on the cyanobacteria that grow on the shells of other individuals and this promotes the growth of mud snails. A similar mutual relationship (as substrate and as food) may be present between *A. cochlephila* and Clausliidae. A species of land snail, *Napaeus barquini* Alonso & Ibáñez, 2006, adheres lichens to its own shell by mucus (Allgaier 2007). This unique behavior is considered a camouflage against predators. Because the molluscous parts are short in the species of Clausliidae examined in this study, it seems impossible for the snails to adhere algae to their shells. However, we cannot rule out the possibility that the shell covered by *A. cochlephila* provides clausliid snails camouflage and thus protection against predators.

The symbiotic relationship between *A. cochlephila* and Clausliidae is not obligate. *A. cochlephila* can grow on artificial media. However, the growth rate of *A. cochlephila* was very low in all culture conditions and media. The addition of a clausliid shell sterilized by dry heat to the medium did not influence the growth rate of *A. cochlephila* (i.e., Namba and Nakayama, unpublished observations). This result may indicate that adequate growth of *A. cochlephila* requires a certain material secreted from living snails or microorganisms inhabiting sympatrically. Further studies are necessary to clarify the relationship between *A. cochlephila* and Clausliidae.

## Electronic supplementary material

Below is the link to the electronic supplementary material.

Supplementary file1 (PDF 666 KB)
