# Correction to: Taxonomic study of a new green alga, *Annulotesta cochlephila* gen. et sp. nov. (Kornmanniaceae, Ulvales, Ulvophyceae), growing on the shells of door snails

**DOI:** 10.1007/s10265-022-01389-6

**Published:** 2022-03-22

**Authors:** Noriaki Namba, Takeshi Nakayama

**Affiliations:** 1 Graduate School of Life and Environmental Sciences, University of Tsukuba, Tsukuba, Ibaraki 305-8572 Japan; 2 Faculty of Life and Environmental Sciences, University of Tsukuba, Tsukuba, Ibaraki 305-8572 Japan

## Correction to: Journal of Plant Research (2021) 134:77–89 10.1007/s10265-020-01239-3

The article "Taxonomic study of a new green alga, Annulotesta cochlephila gen. et sp. nov. (Kornmanniaceae, Ulvales, Ulvophyceae), growing on the shells of door snails", written by Noriaki Namba, Takeshi Nakayama, was originally published Online First without Open Access. After publication in volume 134, issue 1, page 77–89 the author decided to opt for Open Choice and to make the article an Open Access publication. Therefore, the copyright of the article has been changed to © The Author(s) 2021 and the article is forthwith distributed under a Creative Commons Attribution 4.0 International License, which permits use, sharing, adaptation, distribution and reproduction in any medium or format, as long as you give appropriate credit to the original author(s) and the source, provide a link to the Creative Commons licence, and indicate if changes were made. The images or other third party material in this article are included in the article's Creative Commons licence, unless indicated otherwise in a credit line to the material. If material is not included in the article's Creative Commons licence and your intended use is not permitted by statutory regulation or exceeds the permitted use, you will need to obtain permission directly from the copyright holder. To view a copy of this licence, visit http://creativecommons.org/licenses/by/4.0/.

The original article has been updated.

