# Supplementary material for: Taxonomic study of a new green alga, Annulotesta cochlephila gen. et sp. nov. (Kornmanniaceae, Ulvales, Ulvophyceae), growing on the shells of door snails
Source: J Plant Res. 2021 Jan 2;134(1):77–89. doi: 10.1007/s10265-020-01239-3 (PMC8938341; doi:10.1007/s10265-020-01239-3)
Supplement: Supplementary file 1 — Supplementary file1 (PDF 666 KB) [file 10265_2020_1239_MOESM1_ESM.pdf]

Electronic supplementary materials

Title:

Taxonomic study of a new green alga, *Annulotesta cochlephila* gen. et sp. nov. (Kornmanniaceae, Ulvales, Ulvophyceae), growing on the shells of door snails

Authors:

Noriaki Namba & Takeshi Nakayama

Journal:

Journal of Plant Research

Corresponding author:

Noriaki Namba

Graduate School of Life and Environmental Sciences, University of Tsukuba, Tsukuba, Ibaraki 305-8572, Japan

Tel: +81-029-853-4533

Fax: +81-029-853-4533

E-mail: noriaki.nami73@gmail.com

Contents:

Table S1

Fig. S1

Table S1. Sequences used in this study.

**Order**	**Family**	**Sequence name**	**Accession number**
Ulvales	Ulvaceae	*Umbraulva japonica*	AB426255
		Ulvaria obscura	AF499657
		Percursaria percursa	AY303588
		Ulva lactuca	AB425960
		Ulva intestinalis	AF189077
		Ruthnielsenia tenuis	AY454425
		Ochlochaete hystrix	AY454428
	Ulvellaceae	*Ulvella* sp.	AY303593
	Cloniophoraceae	*Cloniophora spicata*	JF680943
	Kornmaniaceae	*Pseudendoclonium arthropyreniae*	MF034609
		Filamentous ulvophyte sp. MBIC10031	AB183574
		Filamentous ulvophyte sp. MBIC10030	AB183573
		Pseudendoclonium incrustans	MF034610
		Pseudendoclonium commune	MF034616
		Pseudendoclonium submarinum	MF034619
		Pseudendoclonium submarinum	EF591129
		Paulbroadya petersii	MF034620
		Paulbroadya prostrata	MF034612
		Photobiont of ascomycete lichen	KM225292
		Halofilum helgolandicum	MF034635
		Halofilum salinum	MF034634
		Halofilum ramosum	MF034615
		Kornmannia leptoderma	AF499661
		Blidingia dawsonii	DQ001138
		Blidingia minima	AF499659
		Uncultured Ulvophyceae clone Alchichica_AL52_5E_36	JN825658

		Lithotrichon pulchrum	MF034614
		Tellamia contorta	AF499663
		Pseudendoclonium fucicola	AF499662
	Bolbocoleonaceae	*Bolbocoleon piliferum*	AY303591
	Ctenocladaceae	*Ctenocladus printzii*	HF570952
		Ctenocladus circinnatus	MF034603
	Phaeopilaceae	*Phaeophila dendroides*	AY454430
Ulotrichales		*Desmochloris mollenhaueri*	FM882217
		Halochlorococcum moorei	AY198122
		Pseudoneochloris marina	U41102
		Acrosiphonia arcta	AY303600
		Capsosiphon groenlandicus	DQ821514
		Monostroma grevillei	AF015279
		Gomontia polyrhiza	AY278216
		Tupiella akinetum	DQ011230
		Ulothrix zonata	MF034653
		Rhexinema planctonica	Z28970
		Chamaetrichon basiliense	Z47996
		Planophila botryoides	AJ416103
		Trichophilus sp. S26_3	GQ462658
		Gayralia sp. ALC-2011	JF680951
Oltmannsiellopsidales	Oltmannsiellopsidaceae	*Neodangemannia microcystis*	AJ416104
		Oltmannsiellopsis viridis	FN562431
Scotinosphaerales	Scotinosphaeraceae	*Scotinosphaera austriaca*	HE860253

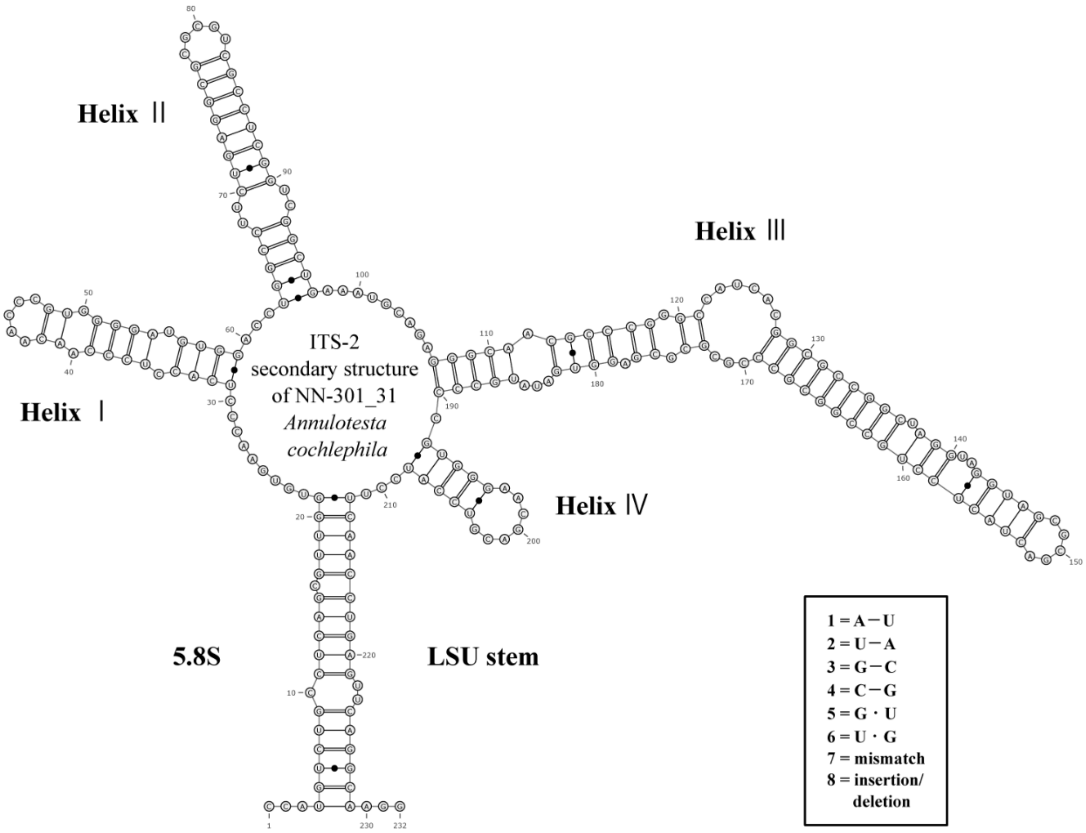

	5.8S / LSU stem	Helix I	Helix II	Helix III
NN-201_8	2364234241332235	641	65344774631	3334145444373433434433413353213
NN-201_9	2364234241332235	641	65344774631	3338145444373433434433413353213
NN-301_31	2364234241332235	641	65344774631	3334145444373433434433413353213
NN-301_32	2364234241332235	641	65344774631	3338145444373433434433413353213
NN-301_34	2364234241332235	641	65344774631	3334145444373433434433413353213
NN-401_2	2364234241332235	641	65364774631	3334145444373433434433413353213
NN-401_4	2364234241332235	641	65364774631	3334145444373433434433413353213
NN-501_41	2364234241332235	641	65364774431	3334145444353433434433413353213
NN-501_42	2364234241332235	641	65364774431	3334145444353433434433413353213
NN-601_10	2364234241332235	641	65364774631	3334145444373433434433413353213
NN-601_11	2364234241332235	641	65364774631	3334145444373433434433413353213
NN-601_12	2364234241332235	641	65364774631	3334145444373433434433413353213
NN-701_1	2364234241332235	641	65364774631	3334145444373433434433413353213
NN-701_2	2364234241332235	641	65364774431	3734147444353433434433413353213
NN-701_3	2364234241332235	641	65344774631	3334145444373433434433413353213
NN-901_1	2364234241332235	641	65366774631	3334145444373433434433413353213
NN-901_2	2364234241332235	641	65364774631	3334145444373433434433413353213
NN-901_3	2364234241332235	641	65366774631	3334145444373433434433413353213
NN-OKN1A_56	2364234241332235	641	65364774631	3336145444373433434433413353213
NN-OKN1A_57	2364234241332235	641	65364774631	3336145444373433434433413353213
NN-OKN1A_58	2364234241332235	641	65364774631	3336145444373433434433413353213
NN-SIG1_51	2364234241332235	641	65344774631	3334145444373433434433413353213
NN-SIG1_52	2364234241332235	641	65364774631	3334145444373433434433413353213
NN-SIG1_53	2364234241332235	641	65364774631	3334145444373433434433413353213
NN-T1_46	2364234241332235	641	65364774631	3334145444373433434433413353213
NN-T1_47	2364234241332235	641	65364774631	3334145444373433434433413353213
NN-T1_48	2364234241332235	641	65364774631	3334145444373433434433413353213

Fig. S1 ITS-2 secondary structure of clone NN301_31 of *A. cochlephila* gen. et sp. nov. The barcode region was translated into a numeric code as described in the Materials and Methods and is provided for each clone sequences in the box below the structure, as in Darienko et al. (2017). Arrow indicates 5 base insertion.
